# Activation of the MAPK pathway is a common event in uveal melanomas although it rarely occurs through mutation of *BRAF* or *RAS*

**DOI:** 10.1038/sj.bjc.6602598

**Published:** 2005-05-31

**Authors:** W Zuidervaart, F van Nieuwpoort, M Stark, R Dijkman, L Packer, A-M Borgstein, S Pavey, P van der Velden, C Out, M J Jager, N K Hayward, N A Gruis

**Affiliations:** 1 Department of Ophthalmology, Leiden University Medical Centre, PO Box 9600, 2300 RC, Leiden, The Netherlands; 2 Department of Dermatology, Leiden University Medical Centre, Wassenaarseweg 72, 2333 AL, Leiden, The Netherlands; 3 Queensland Institute of Medical Research, 300 Herston Rd, Herston, QLD 4029, Australia

**Keywords:** MAPK pathway, uveal melanoma, *BRAF*, *RAS*, mutation

## Abstract

In contrast to cutaneous melanoma, there is no evidence that *BRAF* mutations are involved in the activation of the mitogen-activated protein kinase (MAPK) pathway in uveal melanoma, although there is increasing evidence that this pathway is activated frequently in the latter tumours. In this study, we performed mutation analysis of the *RAS* and *BRAF* genes in a panel of 11 uveal melanoma cell lines and 19 primary uveal melanoma tumours. In addition, Western blot and immunohistochemical analyses were performed on downstream members of the MAPK pathway in order to assess the contribution of each of these components. No mutations were found in any of the three RAS gene family members and only one cell line carried a *BRAF* mutation (V599E). Despite this, mitogen-activated protein kinase/extracellular signal-regulated kinase kinase (MEK), ERK and ELK were constitutively activated in all samples. These data suggest that activation of the MAPK pathway is commonly involved in the development of uveal melanoma, but occurs through a mechanism different to that of cutaneous melanoma.

Uveal melanoma is the most common primary intraocular tumour in adults, with an annual incidence of 6–8 new cases per million among Caucasian populations. Up to half of all patients die from metastatic disease (Diener-West *et al*, 1992). In spite of several known prognostic markers (pathologic and genetic), such as tumour cell type, diameter, localisation and cytogenetic abnormalities, little is known about specific genes associated with predisposition and progression in uveal melanoma (Mooy and de Jong, 1996; Sisley *et al*, 1997). Notable exceptions are hypermethylation of *CDKN2A*, which is more common in tumours from patients who develop metastatic disease (van der Velden *et al*, 2001), and germline *BRCA2* gene mutations, which occur in 3% of patients younger than 50 years of age (Scott *et al*, 2002). Hence, the search for other genes and molecular pathways involved in uveal melanoma development is of great significance. In contrast to uveal melanoma, the influence of specific pathways in cutaneous melanoma, which shares the same embryonic origin, is better defined. For instance, the tumour suppressor gene *PTEN*, encoding a dual-specific phosphatase and a member of the PI3-AKT pathway, plays a major role in the pathogenesis of cutaneous melanoma (Guldberg *et al*, 1997; Stahl *et al*, 2003), whereas no mutations in this gene have been found in uveal melanoma (Naus *et al*, 2000).

Recently, the RAS-RAF-MEK-ERK or mitogen-activated protein kinase (MAPK) pathway has been found to play an important role in melanocytic neoplasia (Cohen *et al*, 2002; Satyamoorthy *et al*, 2003). Activation of this pathway in cutaneous melanocytes has been shown to occur by a variety of mechanisms, including autocrine growth factor stimulation and mutation of the *RAS* or *BRAF* genes. Of three *RAS* genes found to be activated by mutation in human tumours, *NRAS* (neuroblastoma RAS viral (v-*ras*) oncogene homologue) is most commonly mutated in cutaneous melanomas (van Elsas *et al*, 1996). In the active GTP-bound state, RAS activates a number of downstream signalling cascades involved in controlling cell growth and behaviour. Initially, RAS interacts with and activates the serine/threonine protein kinase BRAF that acts in the MAPK pathway to transduce regulatory signals from RAS to mitogen-activated protein kinase/extracellular signal-related kinase kinase (MEK1/2). The signal transducer MEK1/2 phosphorylates extracellular signal-regulated kinase (ERK1/2, p44/42), leading to the activation of these kinases, which in turn activate a variety of transcription factors, including ELK1, again through phosphorylation. It has emerged that *BRAF* (v-*raf* murine sarcoma viral oncogene homologue B1) is very frequently activated by mutation in cutaneous melanomas (Brose *et al*, 2002; Davies *et al*, 2002; Alsina *et al*, 2003; Dong *et al*, 2003; Gorden *et al*, 2003; Kumar *et al*, 2003a, 2003b; Maldonado *et al*, 2003; Omholt *et al*, 2003; Pollock *et al*, 2003; Rimoldi *et al*, 2003; Satyamoorthy *et al*, 2003; Weber *et al*, 2003; Cohen *et al*, 2004; Reifenberger *et al*, 2004; Shinozaki *et al*, 2004; Tsao *et al*, 2004). The frequency of *BRAF* mutations varies from 8 to 83% depending on the anatomic site of the lesion and its histogenic subtype. Notably, the frequency of *BRAF* mutations is also high in benign melanocytic naevi (Dong *et al*, 2003; Pollock *et al*, 2003; Uribe *et al*, 2003; Yazdi *et al*, 2003), indicating that constitutive activation of the MAPK pathway is an early event in melanomagenesis. All *BRAF* mutations in cutaneous pigmented neoplasms occur within the kinase domain. The most frequently found mutation in *BRAF* (V599E) consists of a 1796T → A transversion in exon 15 (Davies *et al*, 2002). Various other mutations have been described in this exon in melanocytic tumours (V599D (Brose *et al*, 2002; Davies *et al*, 2002; Pollock *et al*, 2003); V599K (Pollock *et al*, 2003, Uribe *et al*, 2003); V599R (Pollock *et al*, 2003); K600E (Brose *et al*, 2002, Satyamoorthy *et al*, 2003)). All other mutations have been described in exon 11. The latter consist of a 1352A → C transversion (K438Q) (Brose *et al*, 2002), a 1402G → A transition (G468R) and a 1402/1403GG → TC tandem transversion (Gorden *et al*, 2003), a 1394G → A transition (G465E) and a 1394G → C transversion (G465A) (Davies *et al*, 2002). Furthermore, it is not surprising that since they activate the same pathway, mutations in *NRAS* and *BRAF* are almost mutually exclusive (Brose *et al*, 2002; Davies *et al*, 2002; Alsina *et al*, 2003; Dong *et al*, 2003; Gorden *et al*, 2003; Kumar *et al*, 2003a, 2003b; Omholt *et al*, 2003; Pollock *et al*, 2003; Satyamoorthy *et al*, 2003; Reifenberger *et al*, 2004; Tsao *et al*, 2004).

Since cutaneous and uveal melanoma both arise from neural crest-derived melanocytes, we sought to assess whether the MAPK pathway was similarly activated in melanoma of the uvea. We thus screened for activating mutations in the *NRAS*, *HRAS*, *KRAS* and *BRAF* genes in uveal melanoma cell lines and primary uveal melanomas. Sequence analysis was performed on exons 11–15 of *BRAF*, and exons 1 and 2 of the three *RAS* family members, which cover the positions of all known mutations of these genes in all types of cancer. In addition, we performed immunohistochemistry and Western blot analysis with MEK, ERK and ELK antibodies both on cell lines and/or primary tumours to assess the level of expression and degree of activation of these proteins in order to provide insight into the involvement of this pathway in the development of uveal melanoma.

## MATERIALS AND METHODS

### Cell lines and primary uveal melanoma specimens

In total, 11 uveal melanoma cell lines, derived from primary uveal melanomas (Mel202, Mel 285, Mel 270, Mel 290, Ocm 1, Ocm 3, 92.1, 92.2) or uveal melanoma metastases (Omm 1 Omm 1.3 and Omm 1.5), were analysed. Mel 202, Mel 285, Mel 270, Mel 290 and the two cell lines derived from metastases (Omm 1.3 and Omm 1.5) were kindly provided by Dr BR Ksander (Schepens Eye Institute, Boston, MA, USA). Omm 1, obtained from a subcutaneous metastasis, was established by Dr Luyten (Luyten *et al*, 1996). The cell lines Ocm 1 and Ocm 3 were provided by Dr Kan-Mitchell (Kan-Mitchell *et al*, 1989) and cell lines 92.1 and 92.2, derived from the same primary tumour were established in our own laboratory (de Waard-Siebinga *et al*, 1995). The melanoma cell lines were cultured in RPMI 1640 (Gibco, Paisley, Scotland) medium, supplemented with 3 mM L-glutamine (Gibco), 2% penicillin/streptomycin and 10% FBS (Hyclone, Logan, UT, USA). All cell cultures were incubated at 37°C in a humidified 5% CO_2_ atmosphere. In addition, we analysed 19 primary fresh frozen uveal melanomas. Of the primary tumours, eight were located in the choroid and 11 in both the choroid and ciliary body. Four of these samples showed a spindle cell type, one an epithelioid cell type and 14 had a mixed population of cells. All samples were derived from tumours with a diameter greater than 12 ml and a prominence greater than 6 ml. The research protocol followed the tenets of the Declaration of Helsinki (World Medical Association Declaration of Helsinki 1964; ethical principles for medical research involving human subjects).

### Sequencing

DNA was extracted from each cell line using an adaptation of the salting-out method (Miller *et al*, 1988). Primers used to amplify parts of the *BRAF* and *RAS* genes are given in Table 1.

Reactions for *BRAF* contained 200 ng of DNA, QIAGEN (Hilden, Germany) PCR buffer (10 × concentrated, containing Tris-Cl, KCl, (NH_4_)_2_SO_4_, 15 mM MgCl_2_; pH 8.7), Q solution (PCR enhancer), 20 pmol *μ*l^−1^ of each primer, 2 mM of each dNTP and 1.25 U of QIAGEN *Taq* polymerase. Amplification involved 35 cycles of denaturation at 94°C for 45 s, annealing at 56°C for 90 s, and extension at 72°C for 90 s. An initial 12 min denaturation step at 94°C and a final 3 min extension at 72°C were also used. For the *RAS* genes, DNA was amplified using QIAGEN *Taq* polymerase as described above, but PCR involved a ‘touchdown’ thermal cycling routine of two cycles at each annealing temperature, decreasing by steps of 2°C, followed by 25 cycles at the lowest temperature. Each cycle consisted of denaturation at 94°C for 45 s, annealing at 65–57°C for 90 s and extension at 72°C for 90 s. An initial 12 min denaturation at 94°C and a final 3 min extension at 72°C were also employed.

From 19 fresh frozen uveal melanoma samples, total RNA was extracted with RNeasy kits as described by the manufacturer (QIAGEN). RNA was primed with random primers and reverse transcribed into cDNA in a 20 *μ*l reaction volume containing 200 U Superscript II (MMV) reverse transcriptase (Invitrogen, Inc., Breda, The Netherlands). PCR primers used for amplifying parts of the *BRAF* and *RAS* genes in the primary tumour samples are also listed in Table 1. A touchdown PCR procedure for *BRAF* was followed as described above and a fixed annealing temperature of 57°C with a total of 38 cycles was used for the *RAS* genes followed by a final elongation step of 10 min.

PCR products of all samples were electrophoresed through 1.5% TAE/agarose gels stained with ethidium bromide, excised and purified using a QIAGEN QIAquick Gel Extraction Kit. The *RAS* and *BRAF* PCR products were sequenced using Applied Biosystems (ABI) BigDye version 3 reagents according to the manufacturer's instructions using 3.2 pmol *μ*l^−1^ of primer. Sequencing products were precipitated using 75% isopropanol and were run on an ABI 377 automated sequencer (PE Applied Biosystems, Foster City, CA, USA).

### Western blot analysis

Protein lysates from the uveal melanoma cell lines were separated on 12.5% SDS–PAGE gels and the proteins transferred to Hybond-polyvinyldifluoride membranes (Amersham biosciences, Buckinghamshire, UK). After blocking with 5% skim milk in PBS-Tween solution, the membranes were probed overnight at 4°C with the following primary antibodies specific to each antigen: phospho-MEK1/2 (dilution 1 : 1000), phospho-ERK1/2 (p44/42) (#9106, dilution 1 : 5000), total ERK1/2 (#9102, dilution 1 : 1000) and phospho-ELK1 (dilution 1 : 1000) antibody (all from Cell Signaling Technology, Hertfordshire, UK). An antibody against actin (Santa Cruz Biotechnology, California, USA) was used as a loading control. Membranes were then incubated with horseradish peroxidase-conjugated IgG anti-mouse, anti-rabbit or anti-goat secondary antibodies for 1 h at room temperature to visualise protein bands.

### Immunohistochemistry

Acetone-fixed 10 *μ*m sections of 19 fresh frozen uveal melanomas were washed three times in PBS (pH=7.2) and were incubated with anti-ERK1/2 and anti-phospho-ERK 1/2 antibodies (Cell Signalling Technology, Beverly MA, USA, #9102 and #9106, respectively), both diluted 1 : 100 in PBS with 1% BSA and 2% normal human serum (NHS) at 4°C. The sections were washed three times and incubated with cy3-conjugated AffiniPure goat anti-rabbit IgG or with cy3-conjugated AffiniPure rabbit anti-mouse IgG (Jackson ImmunoResearch, West Grove PA, USA #111-165-003 and #315-165-003) both diluted 1 : 500, respectively, during 1 h at room temperature. Sections were rinsed with PBS three times and incubated for 20 min with Alexa Fluor 647 Phalloidin (Molecular Probes, Leiden, The Netherlands, #A22287) at a 1 : 40 dilution. Sections are washed three times with PBS. A nuclear staining was preformed by incubating the sections for 5 min with 4′,6-diamidino-2-phenylindole dilacetate (DAPI, Molecular Probes, Leiden, The Netherlands, #D-3571) 1 : 500. Sections were rinsed briefly in PBS and imbedded with Vectashield (Vecta Shield H1000, Brunschwig, Amsterdam, The Netherlands). For each specimen, the fluorescence of cy3 was determined in three different microscope fields (Leica DMRXA microscope, Leica Microsystems, Rijswijk, The Netherlands). No background fluorescence of cy3 was observed. The number of positively stained tumour cells was estimated for the two antibodies and expressed as the percentage of the total number of tumour cells in the analysed section. Percentages were then categorised as either negative <5% (+/−), very weakly positive 5–25% (+), weakly positive 26–50% (++), moderately positive 51–75% (+++) or highly positive 76–100% (++++).

The slides were examined by two observers independently. Interobserver disagreement did not exceed one category.

## RESULTS

### Mutation analysis

Of the 11 uveal melanoma cell lines under study, only one cell line (Ocm 1) carried a *BRAF* mutation, the common V599E (also described by Calipel *et al* and Kilic *et al*). All primary tumour specimens were wild type for *BRAF*. No mutations were found in the *NRAS*, *HRAS* or *KRAS* genes, in both the cell lines and primary tissue.

### Western blotting

**Figure 1:**
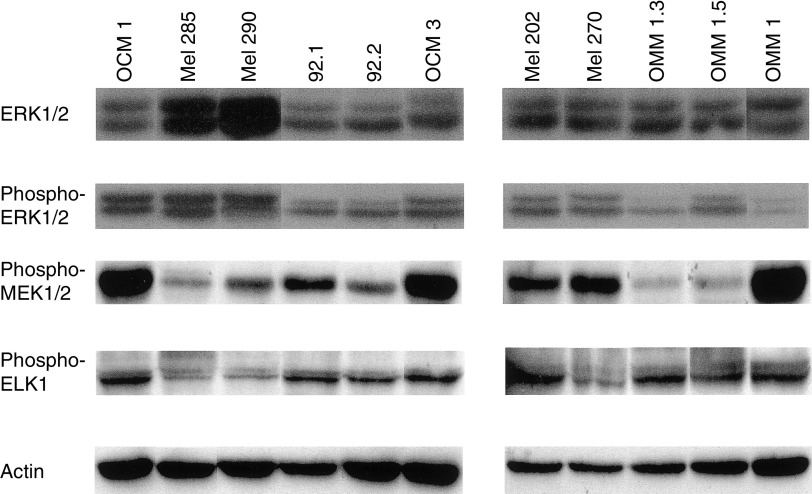
Expression levels of members of the MAPK pathway downstream of RAS and BRAF in 11 uveal melanoma cell lines. Actin levels were assessed as a loading control.

**Figure 2:**
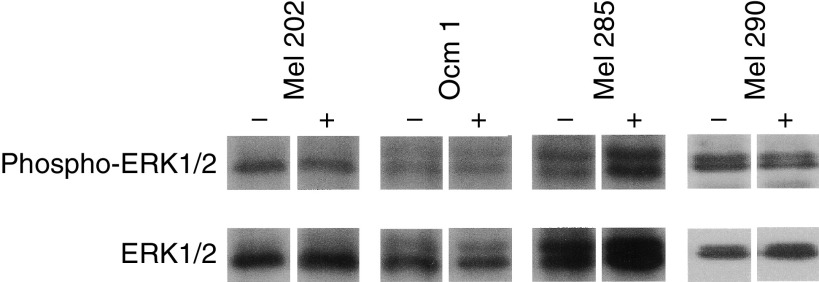
ERK1/2 and phospho-ERK1/2 expression in uveal melanoma cell lines (Mel 202, Ocm 1, Mel 285, Mel 290) cultured with (+) and without (−) serum (24 h). A similar lack of effect of serum on influencing the level of phospho-ERK1/2 was seen for each of the other uveal melanoma cell lines (data not shown).

In order to assess the level of expression and the activation (by phosphorylation) of members of the MAPK pathway downstream of *RAS* and *BRAF*, Western blot analysis was performed on uveal melanoma cell lines (Table 2A). The expression levels of the downstream members of RAS and BRAF are presented in Figure 1. In response to the constitutively activating *BRAF* mutation in Ocm 1, downstream members of the MAPK pathway show activation (phosphorylated MEK, ERK and ELK). Levels of expression of the downstream members were not different in the two cell lines derived from the same primary tumour (92.1 and 92.2), except for phosphorylated MEK, indicating that there had been little clonal divergence between the cell populations during *in vitro* culturing. Interestingly, compared to the phosphorylation status of these members in Ocm 1, most cell lines show activation of MEK, ERK and ELK; however, these cell lines show this activation in the absence of mutations in the upstream *RAS* and *BRAF* genes. The levels of total ERK were remarkably similar across all cell lines, with the exception of two cell lines Mel 285 and Mel 290, which had significantly higher levels of total ERK than the others. In keeping with this observation, these two cell lines also have the highest levels of phosphorylated-ERK. Figure 2 shows that there is no significant influence of serum on the activity of ERK1/2 in these cell lines, as reported recently by Calipel *et al* (2003).

### Immunohistochemistry

Immunofluorescence results of total and phospho-ERK1/2 on a panel of 19 fresh frozen uveal melanoma sections are listed in Table 2 (B). In seven of the 19 primary tumours, less than 5% of the tumour cells stained positively for ERK1/2 and nine tumours for phosphorylated ERK1/2. Despite the lack of mutations in the *RAS* and *BRAF* genes in this set of uveal melanomas, it is noteworthy that we observed phosphorylated (active) ERK1/2 expression in 10 of 19 tumours. There was no significant association between ERK1/2 activation and tumour location or cell type. The scoring system for each antibody cannot be compared between antibodies since the antibodies recognise different epitopes and with different affinities; therefore, the staining intensity on Western or by immunohistochemistry is relative only to the other samples for the particular antibody used.

## DISCUSSION

In the uveal melanoma cell lines and primary uveal melanomas analysed in our study, only cell line Ocm 1 carried a mutation in *BRAF* (V599E), thus confirming the documentation of a mutation in this cell line by Calipel *et al* (2003) and Kilic *et al* (2004). Similarly, our observation of a complete lack of *BRAF* mutations in primary uveal tumours mirrors the findings of several recent reports (Cohen *et al*, 2003; Cruz *et al*, 2003; Edmunds *et al*, 2003; Rimoldi *et al*, 2003; Weber *et al*, 2003; Kilic *et al*, 2004). Table 3 contains a summary of published reports on *RAS* and *BRAF* mutations, as well as studies on other members of the MAPK pathway, in uveal melanomas. Including the results of our study, to date not a single *BRAF* mutation has been found in a total of 276 primary or secondary uveal melanoma samples (Table 3). It is somewhat surprising therefore that three out of three uveal melanoma cell lines studied by Calipel *et al* (2003) carried the V599E mutation in *BRAF*, especially since only one out of 11 cell lines in the panel we analysed was found to have this mutation. Taken together, these data suggest that while a *BRAF* mutation is not required for uveal melanoma development *in vivo*, such mutations confer a cellular growth advantage and are hence selected if they occur in cell lines cultured *in vitro*.

In our study, none of the cell lines or primary tumours carried mutations in any of the three *RAS* genes (N, H and K), a finding consistent with a previous report (Soparker *et al*, 1993). These mutation data are in stark contrast to that for cutaneous melanoma, and would appear to suggest that the MAPK pathway is unlikely to play a significant role in uveal melanoma development. However, on the contrary, by Western blot analysis and immunohistochemistry, we have found substantial evidence for activation of the MAPK pathway, in the absence of serum, in the majority of uveal melanoma samples – both cell lines and primary uveal melanoma specimens. This frequent MAPK pathway activation in uveal melanoma, independent of *RAS* and *BRAF* mutations, has also been reported recently by others (Rimoldi *et al*, 2003; Weber *et al*, 2003;), but the mechanism is unknown. Recently, an interaction has been found between the MAPK and the PTEN pathways, both frequently activated in parallel to promote cutaneous melanoma development (Tsao *et al*, 2004). It is tempting to speculate that MAPK activation in uveal melanoma may arise via crosstalk with the PI3K/PTEN/AKT pathway, possibly as a consequence of mutation of some of its components (other than PTEN, which is not mutated in this tumour type). Thus, mutation analysis of the PI3K and AKT gene families in uveal melanomas seems warranted. Interestingly, Graells *et al* (2004) demonstrated that the proangiogenic vascular endothelial growth factor (VEGF), which is frequently highly expressed in uveal melanoma (Stitt *et al*, 1998; Sheidow *et al*, 2000), could operate in cutaneous melanoma as a survival factor through increasing MAPK and PI3K pathway activity. It is possible that MAPK activation is such a crucial requirement for uveal melanoma development because it similarly provides survival, and/or antiapoptotic signals, necessary for tumour cell growth and maintenance.

Although many uveal melanoma samples have been studied for *BRAF* and *NRAS* mutations, few have been analysed for MAPK activation and there is the implicit assumption that this pathway is not involved in uveal melanoma genesis. Our study is the only study design providing mutation information on all RAS members and expression data on a wide range of participants in the MAPK pathway. Our data thus support the notion that activation of MAPK is indeed involved in the development of uveal melanoma, but occurs via a different mechanism(s) to that in the majority of cutaneous melanomas. This conclusion has significant ramifications for the development of rational therapies to treat uveal melanoma as it implies that general inhibitors of the pathway may still be effective even though the tumours do not have mutations of *RAS* or *BRAF*.

## Figures and Tables

**Table 1:** PCR primers for *BRAF*, *NRAS*, *KRAS* and *HRAS* genes

**Samples**	**Gene**	**Exon/region**	**Primer name**	**Primer sequence (5′>3′)**	**Product size (bp)**
Cell lines	*BRAF*	11	x11F	CTCTCAGGCATAAGGTAATGTAC	
(DNA)			x11R	GAGTCCCGACTGCTGTGAAC	360
	*BRAF*	15	x15F	CTAAGAGGAAAGATGAAGTACTATG	
			x15R	CTAGTAACTCAGCAGCATCTCAG	328
	*NRAS*	1	X1F	CTGGTTTCCAACAGGTTCTTG	
			X1R	TGCTACTCCAATCATCTGGTC	567
	*NRAS*	2	X2F	CACACCCCCAGGATTCTTAC	
			X2R	GTTCCAAGTCATTCCCAGTAG	438
	*HRAS*	1	X1F	GGCAGGAGACCCTGTAGGA	
			X1R	AGCCCTATCCTGGCTGTGT	232
	*HRAS*	2	X2F	AGAGGCTGGCTGTGTGAACT	
			X2R	ACATGCGCAGAGAGAGGACAG	344
	*KRAS*	1	X1F	GATTTTCCTAGGCGGCGG	
			X1R	GTCCGCTCCGTACCTCTCTC	199
	*KRAS*	2	X2F	GGCCTGCTGAAAATGACTG	
			X2R	TATTGTTGGATCATATTCGTCCAC	120
Tumours	*BRAF*	11–15	F	TCAACCACAGGTTTGTCTGC	
(cDNA)			R	GATGACTTCTGGTGCCATCC	696
	*NRAS*		F	GGGGTCTCCAACATTTTTCC	
			R	TCGCTTAATCTGCTCCCTGT	390
	*HRAS*		F	CAGGAGACCCTGTAGGAGGA	
			R	TTTACTGTGATCCCATCTGTGC	968
	*KRAS*		F	AGGCCTGCTGAAAATGACTG	
			R	TTCAATCTGTATTGTCGGATCTC	519

**Table 2:** Summary of mutation analyses, immunohistochemical and Western blotting data in uveal melanoma cell lines and primary uveal melanomas

(A) Uveal melanoma cell lines								
**Cell line**	***BRAF* mutation analysis**	***NRAS* mutation analysis**	***KRAS* mutation analysis**	***HRAS* mutation analysis**	**Total ERK Western**	**Phospho-ERK Western**	**Phospho-MEK Western**	**Phospho-ELK Western**
***From primaries***								
Ocm 1	V599E	WT	WT	WT	+++	+++	++++	+++
Mel 285	WT	WT	WT	WT	++++	++++	++	++
Mel 290	WT	WT	WT	WT	++++	++++	+++	++
92.1	WT	WT	WT	WT	+++	++	+++	+++
92.2	WT	WT	WT	WT	+++	++	++	+++
Ocm 3	WT	WT	WT	WT	+++	++	++++	+++
Mel 202	WT	WT	WT	WT	+++	++	+++	+++
Mel 270	WT	WT	WT	WT	+++	++	+++	++
***From metastases***								
Omm 1.3	WT	WT	WT	WT	+++	+	+	+++
Omm 1.5	WT	WT	WT	WT	+++	+	+	+++
Omm 1	WT	WT	WT	WT	+++	+	++++	+++
(B) Primary uveal melanomas								

**Tumour sample ID**	***BRAF* mutation analysis**	***NRAS* mutation analysis**	***KRAS* mutation analysis**	***HRAS* mutation analysis**	**Total ERK1/2 IHC**	**Phospho ERK1/2 IHC**		
1	WT	WT	WT	WT	++	++++		
2	WT	WT	WT	WT	+++	+++		
3	WT	WT	WT	WT	+	+++		
4	WT	WT	WT	WT	+	+		
5	WT	WT	WT	WT	++	+++		
6	WT	WT	WT	WT	+	++++		
7	WT	WT	WT	WT	+++	+++		
8	WT	WT	WT	WT	+	++		
9	WT	WT	WT	WT	+++	+		
10	WT	WT	WT	WT	++	+		
11	WT	WT	WT	WT	++	+		
12	WT	WT	WT	WT	+++	+++		
13	WT	WT	WT	WT	++	+		
14	WT	WT	WT	WT	+	+		
15	WT	WT	WT	WT	++++	++++		
16	WT	WT	WT	WT	++	+++		
17	WT	WT	WT	WT	+	+		
18	WT	WT	WT	WT	+++	+		
19	WT	WT	WT	WT	++++	+		
Positive control cutaneous melanoma	V599E	NA	NA	NA	+++	++++		

The level of expression of the Western blotting and immunhistochemistry (IHC) experiments were scored and categorised as either negative (–), weakly positive (+), weak–moderately positive (++), moderately positive (+++) or strongly positive (++++).

NA=not applicable.

**Table 3:** Summary of published *RAS* and *BRAF* mutation studies in ocular melanoma

**Study**	**Sample type**	***BRAF* mutation frequency**	***NRAS* mutation frequency**	***KRAS* mutation frequency**	***HRAS* mutation frequency**	**Activation of other MAPK members**
Mooy *et al* (1991)	1° tumours	—	Codons 12,13,61 (0/29)	—	—	—
Soparker *et al* (1993)	1° tumours	—	Exon 1 (0/33)	Exon 1 (0/36)	0/23	—
				Exon 2 (0/39)		
Edmunds *et al* (2003)	1° tumours	0/48	—	—	—	—
Cohen *et al* (2003)	1° tumours	0/29	—	—	—	—
Rimoldi *et al* (2003)	1° tumours	0/10	—	—	—	Expression of MEK/ERK
	2° tumours	0/30				
Weber *et al* (2003)	1° tumours	0/42	0/42	0/42	—	Phospho-ERK (36/42) baseline expression ERK (42/42)
	2° tumours	0/3	0/3	0/3		
Cruz *et al* (Cancer Res.)	Tumours^a^	0/62	Exon 1 (0/22)	—	—	—
			Exon 2 (0/47)			
Calipel *et al* (2003)	Cell lines	3/3^b^	—	—	—	High MEK/ERK levels
Kilic *et al* (2004)	1° tumours	0/33	—	—	—	—
	Cell lines	1/11				
This study	1° tumours	0/19	0/19	0/19	0/19	Activation MAPK pathway: MEK, ELK, ERK
	Cell lines	1/10	0/10	0/10	0/10	

a Not specified if samples were from primary or secondary tumours.

b All V599E.
